# A Closer Look at Anandamide Interaction With TRPV1

**DOI:** 10.3389/fmolb.2020.00144

**Published:** 2020-07-21

**Authors:** Chante Muller, Diane L. Lynch, Dow P. Hurst, Patricia H. Reggio

**Affiliations:** Chemisty and Biochemistry Department, UNC Greensboro, Greensboro, NC, United States

**Keywords:** cannabinoids, TRP channels, anandamide, TRPV1, molecular dynamics

## Abstract

The transient receptor potential subfamily vanilloid type 1 ion channel (TRPV1), located in the peripheral nervous system has been implicated in the perception of pain and possesses the ability to be modulated by various cannabinoid ligands. Because of its location, TRPV1 is an ideal target for the development of novel pain therapeutics. Literature precedent suggests a wide range of cannabinoid ligands can activate TRPV1, but the location and mode of entry is not well understood. Understanding the modes in which cannabinoids can enter and bind to TRPV1 can aid in rational drug design. The first endogenous ligand identified for TRPV1 was the endocannabinoid, anandamide (AEA). The Molecular Dynamics (MD) studies discussed here investigate the entry mode of AEA into TRPV1. During the course of the 10+ microsecond MD simulations, two distinct binding modes were observed: AEA binding in the tunnel formed by the S1–S4 region, and AEA binding in the vanilloid binding pocket, with preference for the former. Unbiased MD simulations have revealed multiple spontaneous binding events into the S1–S4 region, with only one event of AEA binding the vanilloid binding pocket. These results suggest that AEA enters TRPV1 via a novel location between helices S1–S4 via the lipid bilayer.

## Introduction

While CB1 and CB2 are the most commonly known cannabinoid receptors (Howlett, 2002), other receptors and channels have the ability to be modulated by cannabinoid ligands (Akopian et al., 2009; Caterina, 2014). In fact, a subset of transient receptor potential (TRP) channels have been identified as such (Akopian et al., 2009) and have been coined “ionotropic cannabinoid receptors” (Di Marzo et al., 2002). TRP channels are a superfamily of homo- and hetero-tetrameric, transmembrane ion channels involved in the transduction of chemical, mechanical, or physical stimuli to the nervous system (Vay et al., 2012). Topologically, all TRP channels have similar profiles: a tetrameric structure where each monomer has six transmembrane helices, a short pore helix, and a pore loop, with some structural divergences that are characteristic to each class of TRP channels (Gaudet, 2009; Paulsen et al., 2015; Yin et al., 2018). The pore for cation permeation is located through the center of the tetrameric units, with a surface formed by helices 5 and 6 of each monomer. This allows ions to flow from one side of the cell membrane to the other (Gaudet, 2009; Caterina, 2014). TRP channels found in the vanilloid (TRPV1-4), ankyrin (TRPA1), and melastatin (TRPM8) subfamilies can be modulated by various cannabinoid ligands (Muller et al., 2019) and have been located in primary somatosensory neurons (Vay et al., 2012) making them a desirable target for novel pain treatments.

One of these channels, TRPV1, also known as the capsaicin receptor, elicits a burning and tingling sensation upon activation that ultimately leads to desensitization. This process renders the channel refractory to further stimulation, causing a paradoxical analgesic effect. In order to exploit this analgesic effect caused by TRPV1, different avenues of TRPV1 activation and desensitization are being explored, namely by cannabinoid ligands. While vanilloid ligands have been shown via cryo-EM and mutation studies to reside in a binding pocket located between helices 3 and 4 of one monomer and 5 and 6 of an adjacent monomer (Gao et al., 2016), termed the vanilloid binding pocket (VBP), the identity of where cannabinoid ligands bind remains unknown. Due to the identified structural similarities between the endocannabinoid anandamide (AEA) and capsaicin, their similar binding affinities at TRPV1, and similar structural determinants required for sensitivity at TRPV1, it is plausible that AEA and capsaicin could bind in the same location (Ross, 2003). Literature supports the hypothesis that capsaicin gains access to the VBP of TRPV1 by flipping from the extracellular to the intracellular leaflet (Hanson et al., 2015; Yang and Zheng, 2017). However, previously published data shows that the endogenous cannabinoid 2-arachidonoylglycerol (2-AG) enters the cannabinoid CB2 receptor (a G protein-coupled receptor) via the lipid bilayer by passing between two transmembrane helices (Hurst et al., 2010).

A lipid bilayer entry for the endogenous cannabinoid, AEA, into TRPV1 may be different from the entry route for other TRPV1 ligands, such as capsaicin. The major goal of the work described here is to determine how the endogenous cannabinoid, AEA, enters and interacts with TRPV1, using all-atom molecular dynamics (MD) simulations of TRPV1 in a fully hydrated POPC lipid bilayer. Identifying the location of cannabinoid ligand binding to TRPV1 is not only crucial to understanding the mechanism of channel gating, but also provides relevant information that can be used to aid in rational drug design. Since AEA is an endogenous agonist of TRPV1 that can activate the channel, probing the mechanism of binding in a realistic lipid bilayer environment via molecular dynamics simulations is imperative to understanding its role with relation to TRPV1. We find here that there are two distinct binding modes: AEA entering TRPV1 via the tunnel formed by helices S1–S4, and AEA in the VBP. Our results suggest that the preferred mode of AEA entry into TRPV1 is via the tunnel formed by S1–S4 in each monomer of the tetramer.

## Materials and Methods

### Model of Inactive TRPV1

The previously published cryo-EM structure in lipid nanodiscs of TRPV1 in its apo state (PDB: 5IRZ) was used as the template for our model due to its high resolution (3.2 Å) and use of minimal rat sequencing for the transmembrane region of TRPV1 (Gao et al., 2016). The use of the lipid nanodiscs allows the channel to be in an amphipathic environment, largely without disturbing the transmembrane helical structures. A previously crystallized structure of the rat ankyrin repeat domain (ARD) was also used (PDB: 2PNN, 2.7 Å) (Liao et al., 2013). The tetramerized structure of the minimal rat TRPV1 was deconstructed into monomeric subunits and aligned with one ARD. Prime Homology Modeling (Schrödinger, 2018) was utilized to combine these two structures and convert the sequence from rat to human TRPV1 (Jacobson et al., 2004). Due to the high sequence homology of rat and human TRPV1 channels (86%) (Gavva et al., 2004; Johnson et al., 2006) and the lack of bending or kinking residues being introduced into the transmembrane region of the channel, the two resolved structures were aligned and converted with relative ease (Morales et al., 2017). Prime Homology Modeling also allowed for the fulfillment of residues that were absent in the preliminary model, including an extracellular loop of ~25 amino acid residues. A loop refinement was performed using Prime with the OPLS3 force field and VSGB solvation model (Li et al., 2011) at a high dielectric constant to simulate the shape of the loop in an aqueous environment. The completed monomer was then tetramerized and minimized using an implicit membrane from the OPM database, the OPLS3 force field, and the VSGB solvation model to allow the subunits to relax with respect to one another. Note that within the cryo-EM structure(s) published by Gao and colleagues, a lipid headgroup was resolved between helices S1–S4 of the apo TRPV1 structure and was removed prior to the construction of the human TRPV1 model (Gao et al., 2016). Additionally, the authors note a phosphatidylinositol lipid occupying the vanilloid binding pocket of the apo structure. This lipid, suspected to tonically inhibit TRPV1 from constitutive activity, was also removed.

### Unbiased Molecular Dynamic Simulations

**Figure 1:**
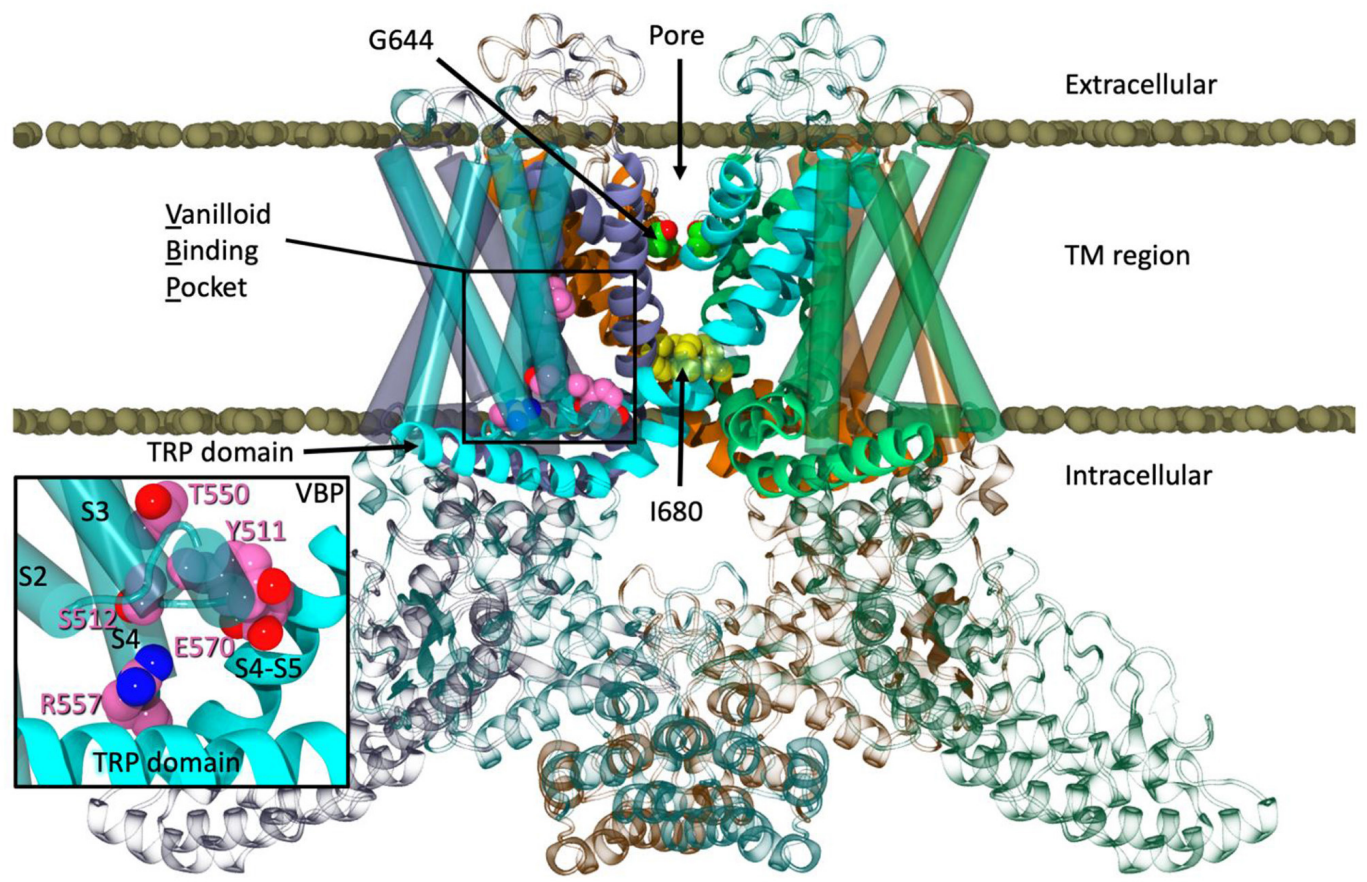
The equilibrated structure of TRPV1 in a POPC lipid bilayer.

For preliminary calculations, the TRPV1 model was embedded in a fully hydrated POPC lipid bilayer with neutralizing ions to bring the ionic strength to 0.15M NaCl. An initial relaxation of the channel was performed following the procedure of Lee et al. (2016). Unbiased NPT MD was performed using CHARMM36 m (Huang et al., 2016) force fields for proteins and CHARMM36 for lipids (Klauda et al., 2010) and ions (Venable et al., 2013) at physiological temperature (310 K) in the fully hydrated lipid bilayer. The CHARMM36 force field for lipids, rather than the OPLS3 force field was used because the CHARMM36 force field is more mature. In order to keep a homogenous force field environment, the CHARMM36 force field was also used for the protein and ligands. A simulation of this system was run using the pmemd.cuda version of AMBER18 (Case et al., 2018) for 500 ns in order to equilibrate the structure (Figure 1). The RMSD of the equilibrated structure and a top-down view of the structure can be found in Supplemental Information, Figures S1B,C, respectively.

During equilibration, water was seen entering a lateral site of TRPV1 located between S1 and S4, which straddles the TRP domain. Further inspection revealed that many polar residues line this region. The cryo-EM structure modeled the density in this region as a lipid headgroup which was removed during hTRPV1 model construction. Additionally, this information, combined with the enhanced flexibility of arachidonic acid derivatives and their entry into CB2 via the lipid bilayer (Barnett-Norris et al., 1998; Hurst et al., 2010), lead us to hypothesize that AEA would have the proper location and flexibility to enter TRPV1 via the tunnel formed by S1–S4, as well as potentially activate the channel from this lateral site.

In order to investigate this, a system was built using a frame from the equilibrated structure of TRPV1 at 50 ns in a 13.8 mol% AEA:POPC fully hydrated lipid bilayer (“Build 1”). Since the structure is homotetrameric, there are four equivalent tunnels that can be observed while this simulation is underway. AEA ligands were randomly dispersed through the upper and lower leaflets of the lipid bilayer. In addition, AEA was placed outside of each tunnel ensuring no incidental contact or interaction occurred with TRPV1 prior to the start of the simulation. This system was run unbiased at 310 K for 642 ns using the pmemd.cuda version of AMBER18.

### Predocked Anandamide in TRPV1

Additional simulations were constructed based on Build 1. One of the noticeable interactions from Build 1 was the ethanolamine headgroup of the AEA interacting with Y554 inside the tunnel of TRPV1 during one of the spontaneous binding events. AEA was docked in all four tunnels, congruent to interactions observed in Build 1, in this second simulation (“Build 2”), and similarly the remainder of the 13.8 mol% AEA was dispersed randomly throughout the upper and lower leaflets of the lipid bilayer. The fully hydrated system was allowed to run unbiased at 310 K using the pmemd.cuda version of AMBER18 for a total of ~370 ns.

### Production Simulations on Anton2

Producing simulations that are microseconds in length for these tetrameric channels embedded in a fully hydrated lipid bilayer is difficult due to their size and complexity. As such, we have continued production molecular dynamics runs on the special purpose supercomputer Anton2 (Shaw et al., 2014) at the Pittsburgh Supercomputing Center. These production simulations were run in the semi-isotropic NPT ensemble at 310 K and 1 bar using the Anton multigrator framework with a Nose-Hoover thermostat and Martyna, Tobias, Klein (MTK) barostat (Nosé, 1984; Hoover, 1985; Martyna et al., 1994). A timestep of 2.5 fs with default Anton settings for the long-range interactions was employed.

## Results

### Unbiased Anandamide Entry Into TRPV1

**Figure 2:**
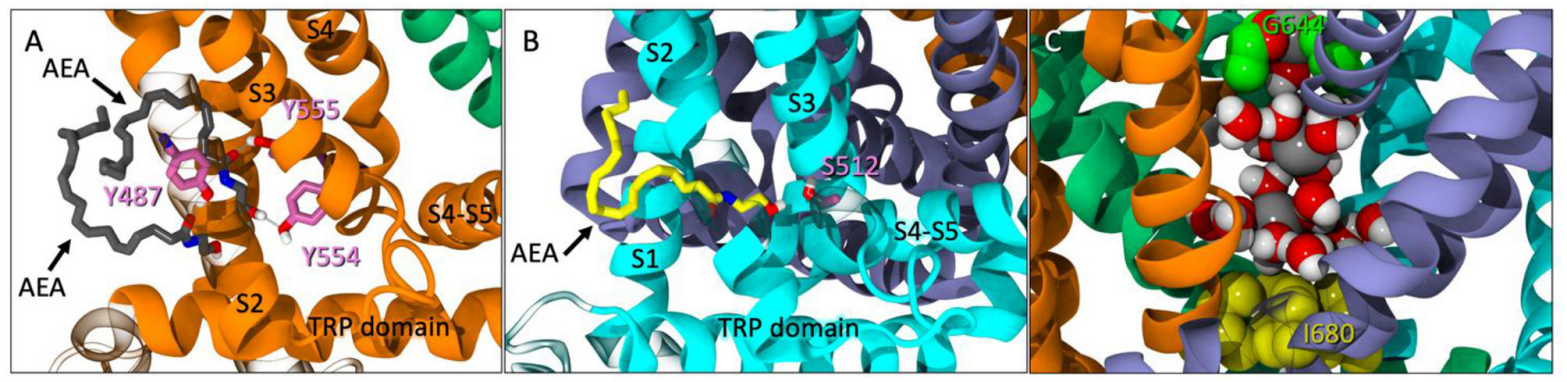
**(A)** Spontaneous entry of two AEA ligands (gray licorice) into one tunnel of TRPV1 at 246 ns. One AEA interacts with both Y554 and Y555 (pink licorice) while the second interacts with Y487 (pink licorice) near the entrance of the tunnel. **(B)** Another instance of spontaneous entry where AEA (yellow licorice) enters the tunnel and interacts with S512 (pink), near the VBP at 315 ns. **(C)** The pore between the upper (G644, green VDW) and lower (I680, yellow VDW) gates with sodium atoms (gray VDW) and water molecules present.

**Figure 3:**
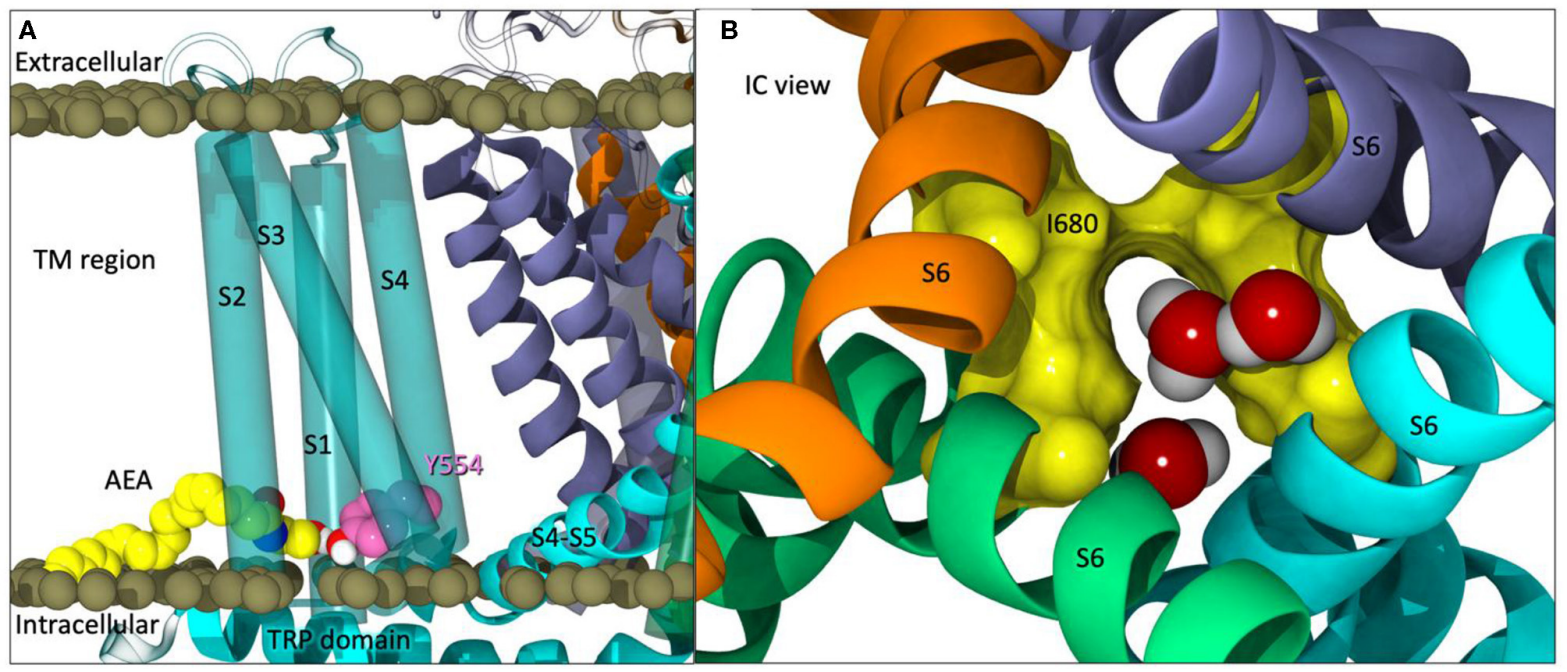
**(A)** The starting point of Build 2 with AEA (yellow) docked in each tunnel with the headgroup interacting with Y554 (pink). **(B)** An intracellular view of partial opening of the lower gate (I680 in yellow surface) with water molecules passing through.

During the unbiased simulation of Build 1, three spontaneous binding events were observed in the tunnels of TRPV1. At one point, two AEA ligands entered the same tunnel, with one AEA interacting with both Y555 and Y554 (Figure 2A). A second AEA enters and settles below the first with the amide oxygen interacting with Y487 near the entrance of the tunnel (Figure 2A). In a separate binding event, a third AEA propelled itself directly into another tunnel, with its headgroup -OH interacting with S512 near the VBP (Figure 2B). Although lower pore opening was not observed during these binding events, the upper pore showed great flexibility (Figure S2D, Supplemental Information), allowing water and sodium ions to fill the pore between the two gates (Figure 2C). It is from these observations that we hypothesized the possibility of AEA entering TRPV1 through the tunnel, and directly or indirectly aiding in the formation of the ionic lock between R557 and E570 that is reported to facilitate gate opening (Gao et al., 2016). This hypothesis was tested in Build 2 (Figure 3A) by docking AEA into each of the tunnels congruent to interactions that were observed during Build 1.

### Increased Gate Flexibility With Predocked Anandamide

Partial opening of the lower gate (I680) was observed after ~125 ns (Figure 3B). At this point in the simulation, one of the four pre-docked AEA ligands have egressed into the lipid bilayer, two other pre-docked AEA ligands backed out of their respective tunnels but still maintain interactions at the entrance of the tunnel, and the final predocked AEA ligand remained stably within its tunnel. When observing the upper gate, G644, the RMSD increases dramatically from 110 to 135 ns (For RMSD see Figure S3C, Supplemental Information), opening at various points within this timeframe. After ~ 140 ns, the upper gate returns to a closed state with small fluctuations. The lower gate undergoes increased fluctuations in the first 10 ns, however after the brief opening at ~127 ns, the lower gate also returns to the closed conformation for the duration of the simulation (For RMSD see Figure S3C, Supplemental Information).

In comparison to our apo simulation, which had no AEA present, no opening of the lower gate was observed. In fact, the lower gate showed incredible stability in comparison to the AEA-containing simulations. The upper gate showed some fluctuations throughout the trajectory allowing water and ions into the pore, however, as noted in Figures 2D, 3C of the Supplementary Information, the frequency at which these events occurred were fewer than those in the presence of AEA.

During the initial simulations (~1.7 μs total), AEA was not observed going into the VBP where vanilloids have been shown to bind. In order to efficiently achieve extended simulation lengths these trajectories were continued on Anton2.

### ANTON2 Simulations Show More Spontaneous Binding

Starting points from Build 1 at 388 ns and Build 2 at 126 ns were selected from the previous simulations to be run on Anton2 for an additional 5.7 microseconds for Build 1 and 6.1 microseconds for Build 2 (Table S1, Supplemental Information).

During the additional microseconds of Build 1 on Anton2, additional spontaneous binding events in the tunnels of TRPV1 were observed throughout the course of the simulation. While the lower gate remained closed, the upper gate showed similar flexibility as observed in the pre-Anton2 builds. In one of the monomers, AEA was shown entering deep into the tunnel and once again interacting with Y544. Conversely, there are relatively few instances of POPC headgroups entering the tunnels during this simulation. In addition, entry into the VBP was not observed.

**Figure 4:**
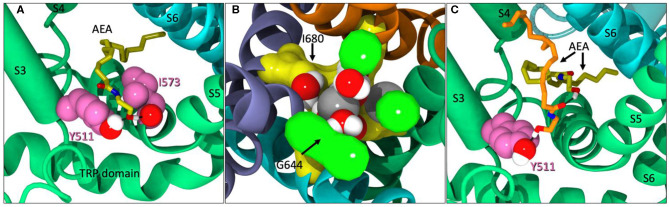
**(A)** AEA (yellow) backing into the VBP while interacting with Y511 and I573. **(B)** The upper gate (G644 in green) of TRPV1 significantly opened. Sodium ions (gray) and water molecules have entered the pore region between G644 and I680 (yellow). **(C)** A second AEA ligand (orange) in the VBP, pushing the first (yellow) back farther into the lipophilic region of the VBP and interacting with Y511.

Within the first microsecond of Build 2 on Anton2, AEA was observed entering the VBP, backing in tail-first, until the -OH headgroup began interacting with Y511 (Figure 4A). At the time of AEA entering the VBP, AEA was also observed occupying three of the four tunnels of TRPV1. The upper gate was significantly opened during this time (Figure 4B). In the microseconds that follow, a second AEA ligand accompanies the first in the lipophilic crevice of the VBP and adjusts itself to now interact with Y511 (Figure 4C). While both of these ligands are simultaneously occupying the same VBP, neither ligand seems to be facilitating the formation of the ionic lock between R557/E570, nor do their interactions appear to increase the flexibility of the lower gate during its occurrence. This might suggest that while AEA binding in the VBP does occur albeit at a lower frequency than in the tunnels, it may not cause activation of the channel.

## Discussion

The complexities and etiologies of chronic pain encompasses many different conditions, symptoms, and pathways, making the condition notoriously difficult to treat. Since cannabis is well known for its analgesic properties, identifying ligands and receptors involved in nociception would greatly benefit the chronic pain population. In chronic pain conditions, action potentials are generated upon stimulation of a nociceptor, propagating the signal to the brain, ultimately resulting in the sensation of pain (Vay et al., 2012). One of the most utilized ways to treat chronic pain conditions currently is with opioid medications. Since the opioid system can influence the reward center, long- and short-term usage can result in addictive behaviors and other unwanted side effects (Storozhuk and Zholos, 2017). However, there is extensive literature precedent that supports the role of cannabinoid ligands, whether phytogenic, endogenous, or synthetic, as modulators of pain largely without the unwanted side effects of opioid medications (Wiese and Wilson-Poe, 2018). This feature coupled with the location of the ionotropic cannabinoid receptors within the peripheral nervous system and their role as sensory transducers provides a potential new target for pain management therapies by targeting that which contributes to the detection of stimuli (Levine and Alessandri-Haber, 2007).

A common example of the role of TRPV1 in pain management is that of capsaicin-based creams. Capsaicin, the pungent compound found in chili peppers, is known to activate TRPV1 and elicits a burning, tingling sensation upon application. However, upon activation, TRPV1 undergoes desensitization which renders the channel refractory to any further stimuli resulting in a paradoxical analgesic effect (Zygmunt et al., 1999; Szallasi et al., 2007; Sanz-Salvador et al., 2012). However, the use of capsaicin and other potent vanilloid agonists like resiniferatoxin (RTX) can have ablative effects on the axon terminals where TRPV1 is located, causing the loss of ability to detect future painful stimuli (Chung and Campbell, 2016). In order to exploit the analgesic effect elicited by TRPV1, different avenues of TRPV1 activation and desensitization are being explored, namely by cannabinoid ligands.

While the binding modes of vanilloid ligands like capsaicin and resiniferatoxin have been well studied via cryo-EM and mutation data, the binding mode of cannabinoid ligands at TRPV1 has yet to be studied at the same level of detail. Throughout this series of MD simulations, totaling over a collective 10 microseconds, two distinct binding modes were observed: a novel point of entry in which AEA enters into the tunnel located between helices S1–S4, as well as AEA entering the putative VBP. Of these two modes, the binding pathway that was most prevalent in our simulations was that of AEA entering into the tunnels of TRPV1, formed by helices S1–S4, via the lipid bilayer. Additionally, the frequency at which AEA enters the tunnels of TRPV1 spontaneously far exceeds that of AEA entering the VBP. Thirteen unique AEA ligands were found to spontaneously enter into the tunnels of TRPV1 throughout the trajectory of Build 1, suggesting a low energy barrier for AEA entry at this location. In contrast, only one instance of AEA ligands entering the VBP directly was observed throughout the 10+ microseconds of simulation time. It is also worth noting that the system in which VBP binding occurred (Build 2), had all S1–S4 tunnels of TRPV1 occupied with AEA, whether fully inserted into the tunnel or interacting with residues near the entrance.

While the entrance of AEA into the tunnel region of TRPV1 is not the putative location, it is not unusual for TRPV channels to have several allosteric sites. In the cryo-EM structures published by Gao, et. al, a spider toxin called Double Knot Toxin was shown to bind to and activate TRPV1 via the extracellular side of the channel (Gao et al., 2016). Recently, cannabidiol (CBD) has been resolved in TRPV2 between helix 5 of one monomer and helix 6 of an adjacent (Pumroy et al., 2019). This region in TRPV2 has high sequence homology with other TRPV channels, potentially indicating that CBD could interact with TRPV1 at the same location. These varieties to ligand binding at TRPV1 beyond the VBP lend credence to the idea that in addition to its many modes of activation, TRPV1 also has more than one site that can be occupied by a ligand. Additionally, previously published data which shows that the endogenous cannabinoid 2-arachidonoylglycerol (2-AG) enters the CB2 receptor between two transmembrane helices via the lipid bilayer (Hurst et al., 2010) supports our finding that AEA entry into TRPV1 occurs in a similar fashion. Since both 2-AG and AEA contain an arachidonic acid tail, they both possess great flexibility, allowing it heightened mobility within the lipid bilayer, allowing it to reach regions of the protein that might be inaccessible to other ligands.

While sustained opening of the lower gate was not observed during these initial unbiased multi-microsecond simulations, opening of the upper gate was sampled frequently on multiple occasions, allowing water and ions to enter the pore. This was anticipated due to the location of the upper gate being in a loop region and formed by four glycine carbonyl oxygen atoms which appear to coordinate with a sodium ion. The opening of the upper gate allowed water and ions to enter the pore region between the two gates, and while complete opening of the lower gate has not yet been achieved, partial opening was observed in the predocked AEA system. In the apo build, the lower gate was very stable throughout the entirety of its trajectory, showing only mild flexibility. Additionally, the tunnels of the apo run remained largely unoccupied in the absence of AEA, while the AEA containing systems showed multiple binding events, including spontaneous binding from a completely unbiased system, as well as, the exit and re-entry of AEA ligands in the predocked system. The results of these simulations suggest that AEA prefers the lateral site of TRPV1 over the VBP. Due to the frequency of AEA interacting with Y554 and Y555 in the S1–S4 tunnel, it is possible that mutating these residues to phenylalanine to remove hydrogen bonding capability, or mutating to alanine, to remove both hydrogen bonding and aromatic stacking interactions, may alter or ablate AEA binding in this location. While this is speculation, the data from these simulations suggest that both Y554 and Y555 play a role in AEA interaction with the S1–S4 tunnel.

Regardless of the structural similarities between capsaicin and AEA, our simulations suggest that AEA can enter TRPV1 via the S1–S4 tunnel with a higher probability than that of the VBP. The simulations discussed here are a promising start to better understanding the interactions between AEA and TRPV1 on a molecular level and introduces the idea that AEA enters and interacts with TRPV1 in a novel location between helices S1–S4.

## Data Availability Statement

All datasets presented in this study are included in the article/Supplementary Material.

## Author Contributions

All authors listed have made a substantial, direct and intellectual contribution to the work, and approved it for publication.

## Conflict of Interest

The authors declare that the research was conducted in the absence of any commercial or financial relationships that could be construed as a potential conflict of interest.
