# Supplementary material for: A Closer Look at Anandamide Interaction With TRPV1
Source: Front Mol Biosci. 2020 Jul 21;7:144. doi: 10.3389/fmolb.2020.00144 (PMC7385410; doi:10.3389/fmolb.2020.00144)
Supplement: Supplementary file 1 [file Table_1.DOCX]

Supplemental Material


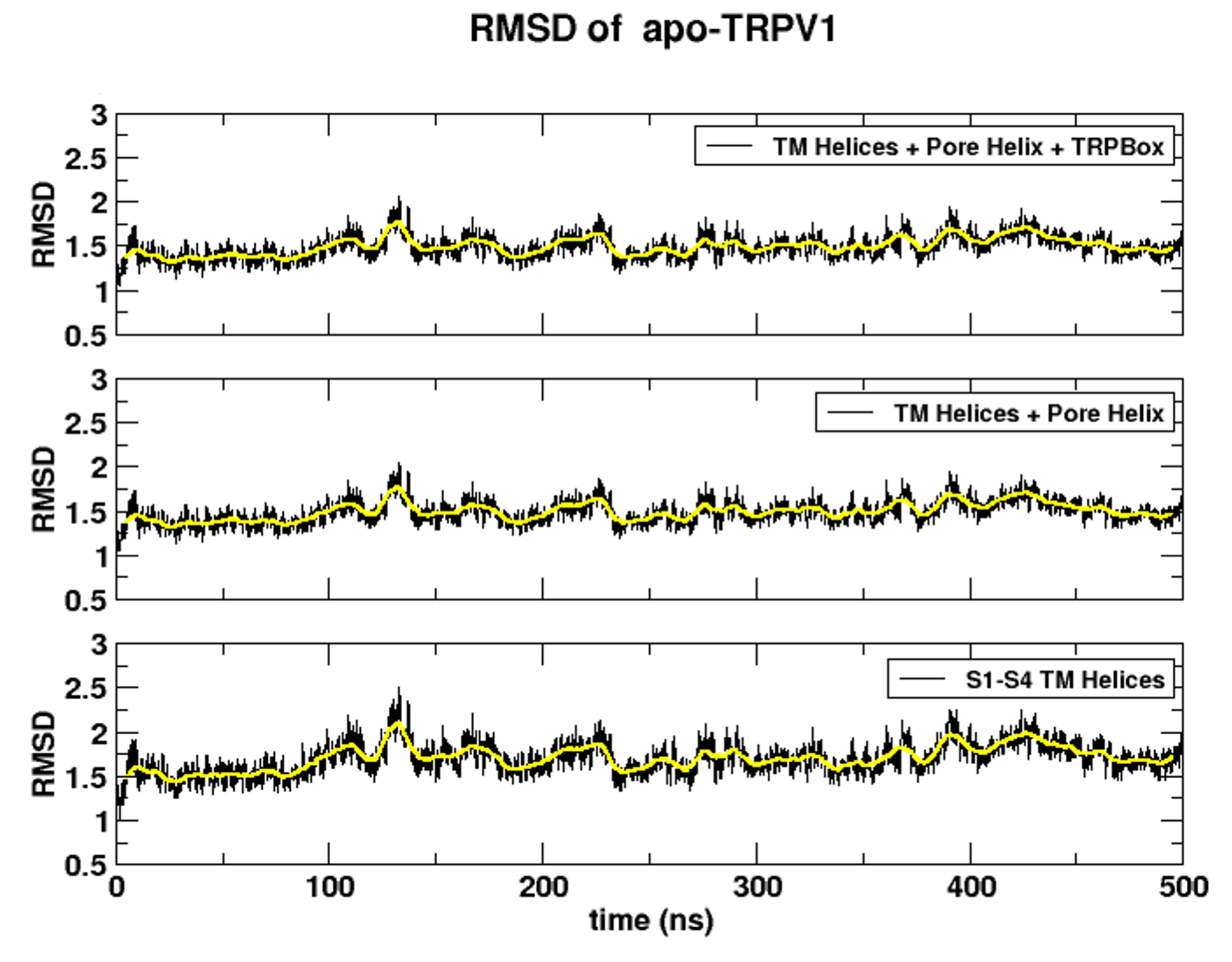


Figure 1b. The RMSD of apo-TRPV1 structure over the course of 500ns to show the stability of the model. The top panel shows the RMSD the transmembrane (TM) region (residues 430 to 455, 474 to 497, 510 to 532, 536 to 551, 576 to 597, and 656 to 687), the pore helix (residues 633 to 643), and the TRP box (residues 892 to 711). The middle panels shows the RMSD of the TM helices and pore helix. The last panel shows the RMSD of the TM helices only.


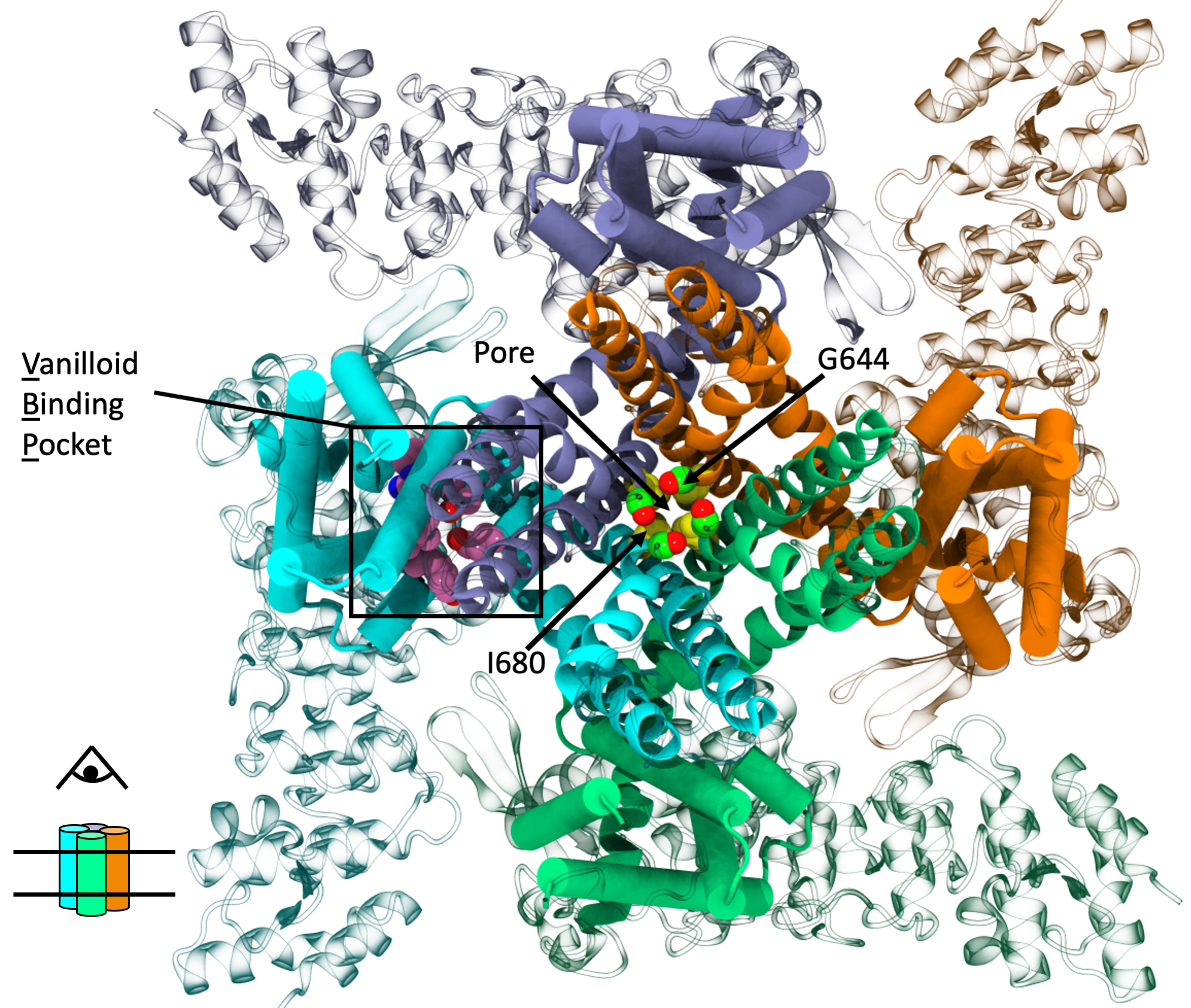


Figure 1c. A top-down view of TRPV1.

Table 1:

| System | Ligand | Initial Conformation | Length |
| --- | --- | --- | --- |
| Apo | None | Human model from cryo-EM | 500ns |
| Build_1 | 13mol% AEA | Human model from cryo-EM | 642ns |
| Build_2 | 13mol% AEA | Human model from cryo-EM | 370ns |
| Build_1 on ANTON2 | 13mol% AEA | 388ns from Build_1 | 5.7μs |
| Build_2 on ANTON2 | 13mol% AEA | 126ns from Build_2 | 6.1μs |


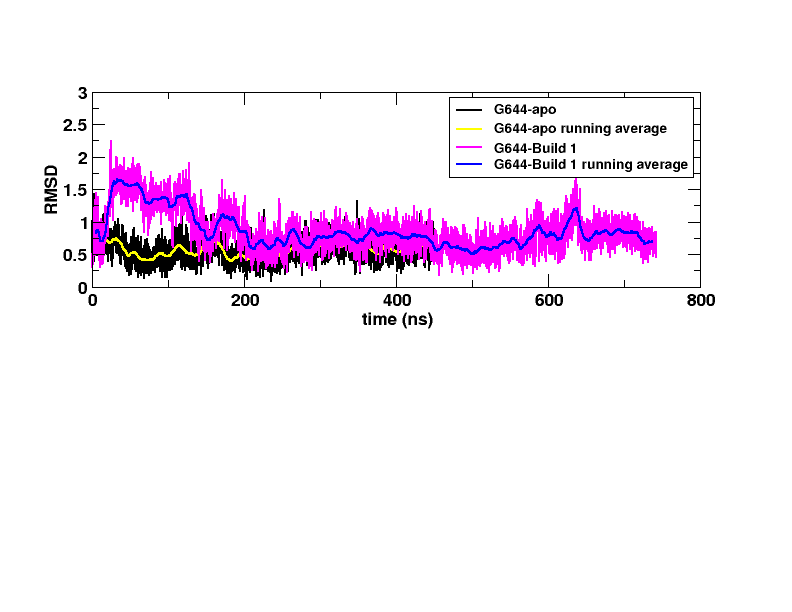


Figure 2D. The RMSD of the upper gate, G644, of the control shown in black with the average shown in yellow against the RMSD of the upper gate, G644, of Build 1 shown in magenta with the average shown in blue.


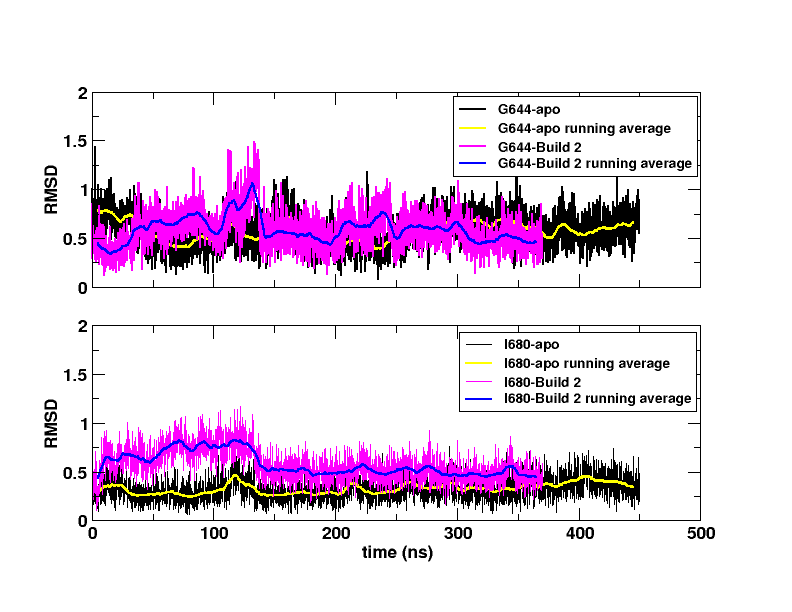


Figure 3C. Upper panel shows the RMSD of the upper gate, G644, in the control run (G644-apo) with the average RMSD shown in yellow. The RMSD of the upper gate from Build 2 is shown in magenta with the average RMSD shown in blue. Lower panel shows the RMSD of the lower gate, I680, in the control run (I680-apo) with the average RMSD shown in yellow. The RMSD of the lower gate from Build 2 is shown in magenta with the average RMSD shown in blue.
